# Mutational characterization of Omicron SARS-CoV-2 lineages circulating in Chhattisgarh, a central state of India

**DOI:** 10.3389/fmed.2022.1082846

**Published:** 2023-01-23

**Authors:** Pushpendra Singh, Kuldeep Sharma, Dipika Shaw, Anudita Bhargava, Sanjay Singh Negi

**Affiliations:** Department of Microbiology, All India Institute of Medical Sciences, Raipur, Chhattisgarh, India

**Keywords:** COVID-19, SARS-CoV-2, Omicron, BA.2.75, BA.2.38, BA.5

## Abstract

**Introduction:**

The emergence of the Omicron SARS-CoV-2 variant from various states of India in early 2022 has caused fear of its rapid spread. The lack of such reports from Chhattisgarh (CG), a central state in India, has prompted us to identify the Omicron circulating lineages and their mutational dynamics.

**Materials and methods:**

Whole-genome sequencing (WGS) of SARS-CoV-2 was performed in 108 SARS-CoV-2 positive combined samples of nasopharyngeal and oropharyngeal swabs obtained from an equal number of patients.

**Results:**

All 108 SARS-CoV-2 sequences belonged to Omicron of clade 21L (84%), 22B (11%), and 22D (5%). BA.2 and its sub-lineages were predominantly found in 93.5% of patients, BA.5.2 and its sub-lineage BA.5.2.1 in 4.6% of patients, and B.1.1.529 in 2% of patients. Various BA.2 sub-lineages identified were BA.2 (38%), BA.2.38 (32%), BA.2.75 (9.25%), BA.2.56, BA.2.76, and BA.5.2.1 (5% each), BA.2.74 (4.6%), BA.5.2.1 (3.7%), BA.2.43 and B.1.1.529 (1.8% each), and BA.5.2 (0.9%). Maximum mutations were noticed in the spike (46), followed by the nucleocapsid (5), membrane (3), and envelope (2) genes. Mutations detected in the spike gene of different Omicron variants were BA.1.1.529 (32), BA.2 (44), BA.2.38 (37), BA.2.43 (38), BA.2.56 (30), BA.2.74 (31), BA.2.75 (37), BA.2.76 (32), BA.5.2, and BA.5.2.1 (38 similar mutations). The spike gene showed the signature mutations of T19I and V213G in the N-terminal domain (NTD), S373P, S375F, T376A, and D405N in receptor-binding domain (RBD), D614G, H655Y, N679K, and P681H at the furin cleavage site, N764K and D796K in fusion peptide, and Q954H and N969K in heptapeptide repeat sequence (HR)1. Notably, BA.2.43 exhibited a novel mutation of E1202Q in the C terminal. Other sites included ORF1a harboring 13 mutations followed by ORF1b (6), ORF3a (2), and ORF6 and ORF8 (1 mutation each).

**Conclusion:**

BA.2 followed by BA.2.38 was the predominant Omicron lineage circulating in Chhattisgarh. BA.2.75 could supersede other Omicron due to its mutational consortium advantage. The periodical genomic monitoring of Omicron variants is thus required for real-time assessment of circulating strains and their mutational-induced severity.

## 1. Introduction

SARS-CoV-2 B.1.1.529, better known as “Omicron” and designated as a variant of concern (VOC), was first reported from Botswana and South Africa on 11 and 14 November 2021, respectively (1, 2). Omicron has speedily surged globally in around 171 countries, including India to out-number other VOCs such as Alpha (B.1.1.7), Beta (B.1.351), Gamma (P.1), and Delta (B.1.617.2) (1, 2).

The Omicron variant’s high mutational divergence, especially in spike glycoprotein, has been attributed to higher transmissibility and evading the vaccine-induced immune response. Omicron thus also poses an imminent global threat to our effort to achieve protection against COVID-19 through mass vaccinations. Most vaccines and therapeutics used in treatment and prevention were based on spike protein to prevent the virus from attaching to host cell receptor angiotensin-converting enzyme 2 (ACE 2) before the viral infection ensues. The hypervariable mutations in the spike gene further diversify Omicron to five sub-lineages (BA.1, BA.2, BA.3, BA.4, and BA.5), lineages, and subvariants, posing a further threat of COVID-19 outbreak (3). In India, with the surge of BA.2 Omicron sub-lineages, many reinfections have been observed to raise the apprehension of whether BA.2 precisely escapes the natural immunity acquired after a previous COVID-19 infection (4). In India, Omicron transmission has continuously been reported from December 2021 till the time of reporting this study in October 2022. During the said period, it evolved from sub-lineages B.1.1.529 to BA.2, BA.5.2, and their various sub-lineages to become the predominant strain (5). However, there is no report from the state of Chhattisgarh in central India about the Omicron variant and its sub-lineages circulating among infected COVID-19 cases.

Accordingly, this study was undertaken to perform WGS of SARS-CoV-2 strains isolated from patients with COVID-19 reported at various districts hospitals across Chhattisgarh to stratify SARS-CoV-2 Omicron lineages and sub-lineages, their mutational pattern, and potential effects, primarily on the spike glycoprotein.

## 2. Materials and methods

This study was performed at the State-Level Viral Research and Diagnostic Laboratory (VRDL), All India Institute of Medical Sciences (AIIMS), Raipur, Chhattisgarh, a designated state nodal tertiary care treatment and diagnostic center for COVID-19 and a member of the Indian SARS-CoV-2 Genomics Consortium (INSACOG), for genomic surveillance of SARS-CoV-2.

### 2.1. Sample collection

Under the national Integrated Disease Surveillance Program (IDSP) genomic monitoring surveillance, the combined clinical specimen of the nasopharyngeal and oropharyngeal swabs in viral transport medium was collected between 1 May and 13 July 2022 from 270 laboratory-confirmed cases of COVID-19. These samples were then transported under cold conditions to the state Viral Research Diagnostic Laboratory (VRDL), AIIMS, Raipur. These 270 cases demographically belonged to Raipur (110), Bilaspur (92), Surguja (29), Raigarh (13), Baloda Bazar (9), Durg (6), Jagdalpur (10), and Mahasamund (1). All these cases were clinically reported as showing mild upper respiratory tract infection and recovered completely without hospitalization. The clinical samples were first processed for the qualitative detection of SARS-CoV-2 using the ICMR-NIV manufactured Real-Time Polymerase Chain Reaction (RT-PCR) kit as described earlier to confirm COVID-19 infection (6). All samples were positive, yet only 108 showed Ct values less than 25 for the E and RdRp genes of SARS-CoV-2. They were only processed for WGS of SARS-COV-2 after obtaining institutional ethics approval (1453/IECAIIMSRPR/2021). These 108 samples demographically represented the patients from Raipur (75), Bilaspur (20), Durg (4), Jagdalpur and Raigarh (3 each), and Baloda Bazar, and Mahasamund (1 each).

### 2.2. SARS-CoV-2 whole-genome sequencing

Whole-genome sequencing of the SARS-CoV-2 virus from 108 clinical samples was performed as described previously (5). Briefly, the isolated RNA from every sample was first converted into cDNA. All cDNAs were then processed for library preparation using the QIAseq DIRECT SARS-CoV-2 Enhancer kit and the QIAseq FX DNA Library Unique Dual Index (UDI) kit from Qiagen GmbH, Germany. The SARS-CoV-2 whole-genome sequencing was performed in Illumina MiniSeq sequencers using Mid Output Reagent Cartridge (300 cycles) in 150 × 2 PE read and FastQ mode using MiniSeq local run manager. The whole-genome sequences of 108 SARS-CoV-2 were curated and analyzed using CLC Genomics Workbench version 21. The sequences were successfully submitted in the GISAID, wherein the identifier number EPI_SET_221213kr was provided.

### 2.3. Phylogenetic and mutational analyses

The structural and non-structural protein-coding gene sequences were aligned with reference Wuhan-Hu-1 (GenBank accession number: NC_045512) sequence by using the bioinformatics tool MAFFT version 7.310 (7). The phylogenetic tree was constructed using the neighbor-joining (NJ) method and the Kimura 2-Parameter model of MEGA7 software (8). A bootstrap re-sampling process with 1,000 replications assessed the robustness of individual nodes. Interactive Tree Of Life version 6 (iTOLv6) was used for the phylogenetic tree display and annotation (9). Lineage-specific mutation prevalence was derived from the analysis of consensus genomes. The nucleotide insertion/substitutions/deletions were identified to prepare a heat map.

## 3. Results

Demographic analysis revealed 108 cases comprised of 57 (53%) men and 51 (47%) women. The collective mean age was 37.43 years for both men and women, while it was 39.47 years for men and 35.13 years for women.

**FIGURE 1:**
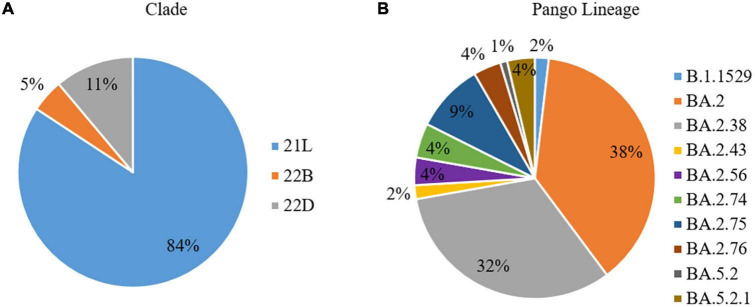
Spectrum of the whole-genome sequences of SARS-CoV-2 (*N* = 108), in terms of **(A)** Clades and **(B)** Pango Lineage, from patients of the state of Chhattisgarh.

**FIGURE 2:**
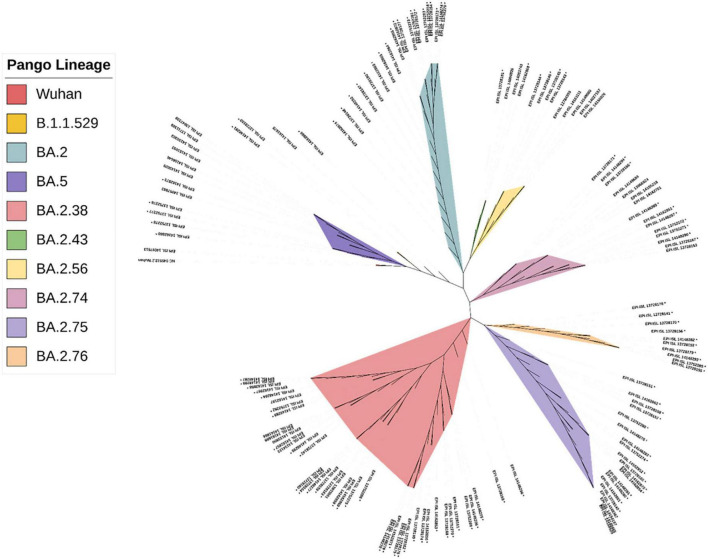
The phylogenetic distribution of whole-genome sequences of 108 SARS-CoV-2 strains from Chhattisgarh, India, and 30 samples from elsewhere, with the reference sequence of the Wuhan strain.

**FIGURE 3:**
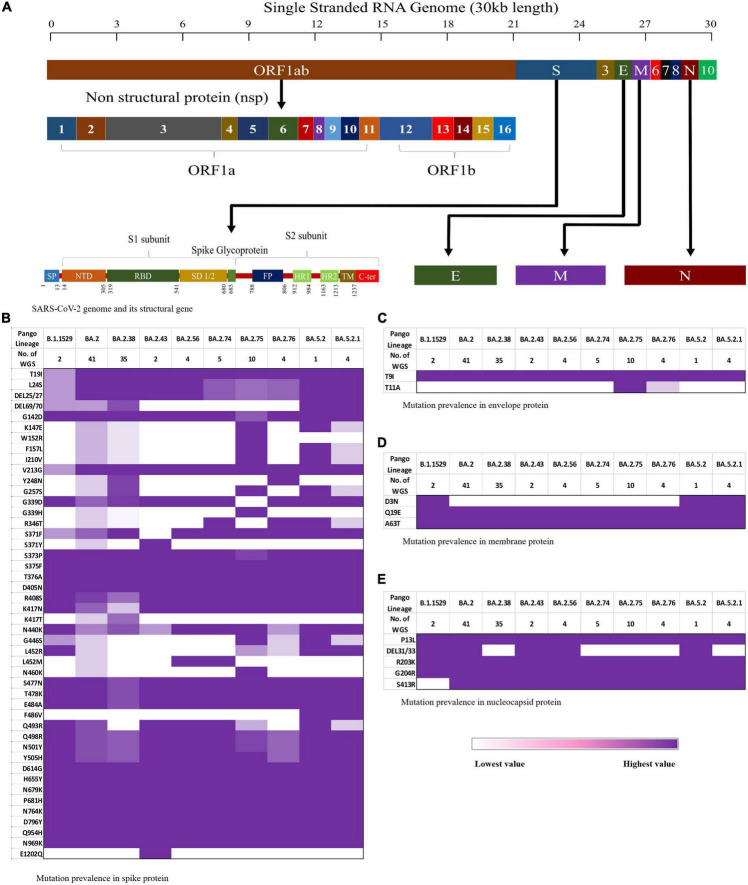
Schematic representation of the mutation pattern in the four structural proteins is shown as a heat map from lowest (white) to highest (purple). **(A)** The SARS-CoV-2 genomic arrangement, **(B)** mutation pattern in spike protein, **(C)** mutation pattern in envelop protein, **(D)** mutation pattern in membrane protein, and **(E)** mutation pattern in nucleocapsid protein.

The CLC Genomics Workbench showed average sequence quality of 95–99%. The Nextclade software analysis of 108 sequences of SARS-CoV-2 revealed 91 (84%) sequences belonged to clade 21L, 12 (11%) sequences to clade 22B, and five (5%) sequences to clade 22D (Figure 1A) (10). The unrooted phylogenetic tree analysis of these sequences with Wuhan reference NC_045512 sequence and 30 representative sequences of different lineages revealed all 108 SARS-CoV-2 variants belonging to Omicron and their different sub-lineages (Figure 2). Apart from two B.1.1.529 sequences (2%), the majority of 101 sequences (93.5%) belonged to BA.2 and its sub-lineages, while five sequences (4.6%) identified to BA.5.2 and its sub-lineage BA.5.2.1. Among the BA.2 lineages, BA.2, BA.2.38, BA.2.43, BA.2.56, BA.2.74, BA.2.75, and BA.2.76 were detected in 40 (^~^38%), 35 (^~^32%), 2 (^~^2%), 4 (^~^4%), 5 (^~^4%), 10 (^~^9%), and 4 (^~^4%) sequences, respectively. In the remaining five sequences, BA.5.2 was found in one sequence (^~^1%), while BA.5.2.1 was found in four cases (^~^4%) (Figure 1B). The WGS analysis revealed 79 mutations, with the majority of 46 detected in spike glycoprotein (Figure 3). Other mutational sites observed among structural genes were envelope, membrane, and nucleocapsid, while in non-structural protein (NSP), mutations were found in NSP1-11 encoding open reading frame (ORF) 1a, NSP12-16 encoding ORF1b, ORF3a, ORF6, and ORF8, respectively (Supplementary Table 1).

**FIGURE 4:**
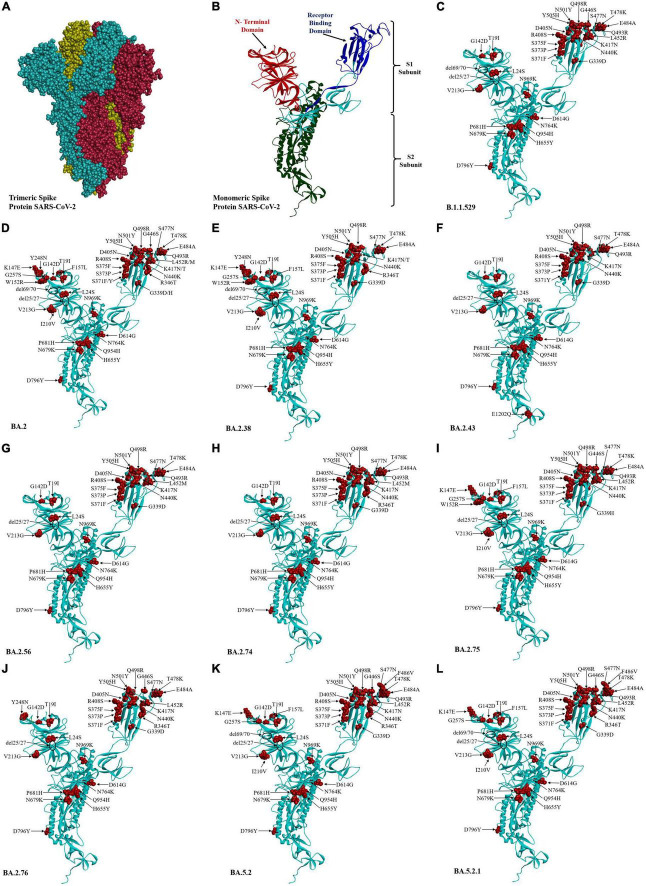
Three-dimensional structure and amino acid residue mutations in the spike protein of SARS-COV-2 Omicron variant and their sub-lineages. Wuhan trimeric spike glycoprotein prototype **(A)** and monomeric spike glycoprotein prototype **(B)** in which red, blue, and green colors showed the N-terminal domain, receptor-binding domain, and S2 subunit of spike proteins, respectively. The amino acid residue substitution and deletion sites are marked in red color and circle, respectively, in Omicron sub-lineages **(C–L)**.

The three-dimensional structure of the Wuhan prototype of SARS-CoV-2 spike protein in homotrimeric and monomeric form was used as a reference to analyze the mutational effect (Figures 4A, B). A maximum of 44 mutations were analyzed in BA.2 followed by 38 mutations seen in BA.2.43, BA.5.2, and BA5.2.1 (albeit at different amino acids), BA.2.38 and BA.2.75 (37 mutations each), BA.2.76 (32 mutations), BA.2.74 (31 mutations), and BA.2.56 (30 mutations) (Figure 4 and Table 1). Notably, 25 mutations were seen in the receptor-binding domain (RBD) followed by 12 mutations in the NTD region in varying proportions among different Omicron lineages. Importantly, sub-domain (SD) 1, SD2, furin cleavage site, fusion peptide (FP), and heptapeptide repeat sequence (HR1) region were observed with a total of eight common mutations among all the detected Omicron lineages (Table 1). BA.2.43 exhibited a unique mutation of E1202Q in the HR2 region of spike protein, while BA.2.75 showed exclusive mutation of N460K (Figure 3 and Table 1). The worth noticing in the spike gene was signature mutations of T19I and V213G in the N-terminal domain (NTD), S373P, S375F, T376A, and D405N in RBD, D614G, H655Y, N679K, and P681H at the furin cleavage site, N764K and D796K in fusion peptide, and Q954H and N969K in heptapeptide repeat (HR)1 among all the detected lineages.

**TABLE 1:** Omicron different lineages mutational pattern in spike protein.

Lineage	Total mutation	Region		Mutation (No.)	Mutational pattern
B.1.1.529	32 (2 Deletion, 30 AA substitution)	S1	NTD	6	T19I, L24S, Δ25/27, Δ69/70,G142D, V213G
			RBD	18	G339D, S371F, S373P, S375F, T376A, D405N, R408S,G446S, S477N, T478K, E484A, Q493R, L452R, K417N, N440K, Q498R, N501Y,Y505H
			SD1 and 2*	3	D614G, H655Y, N679K,
			Furin CS*	1	P681H
		S2	FP*	2	N764K, D796Y
			HR1*	2	N954H, N969K
BA.2	44 (2 Deletion, 42 AA substitution)	S1	NTD	12	NTD of B.1.1.529 + K147E, W152R, F157L,I210V, Y248N,G257S
			RBD	24	RBD of B.1.1.529 + G339H, R346T, S371Y,K417T, L452M, N460K
BA.2.38	37 (2 Deletion, 35AA substitution)	S1	NTD	12	Same as BA.2
			RBD	17	Alteration in B.1.1.529 of K417N/T, new substitution R346T, and absence of G446S, L452R, Q493R
BA.2.43	30 (1 Deletion, 28 Substitution)	S1	NTD	5	Same as B.1.1.529 with absence of Δ69/70
			RBD	16	Alteration in B.1.1.529 of S371Y and absence of G446S, L452R
		S2	HR2	1	E1202Q
BA.2.56	30 (1 Deletion, 28 Substitution)	S1	NTD	5	Same as B.1.1.529 with absence of Δ69/70
			RBD	17	Alteration in B.1.1.529 of L452M and absence of G446S
BA.2.74	31 (1 Deletion, 30 Substitution)	S1	NTD	5	Same as B.1.1.529 with absence of Δ69/70
			RBD	18	Alteration in B.1.1.529 of L452M and new mutation of R346T, absence of G446S
BA.2.75	37 (1 Deletion, 36 Substitution)	S1	NTD	10	Same as BA.2 with absence of Δ69/70, Y248N
			RBD	19	Alteration in B.1.1.529 of G339H and new mutation of N460K
BA.2.76	32 (1 Deletion, 31 Substitution	S1	NTD	6	Same as BA.2.74 with new mutation of Y248N
			RBD	18	Same as B.1.1.529 with absence of Q493R and new mutation of R346T
BA.5.2 and BA.5.2.1	38 (2 Deletion, 31 Substitution)	S1	NTD	10	Same as BA.2 with absence of W152R, Y248N
			RBD	20	Same as BA.2.76 with new mutation of F486V, Q493R

HR, heptapeptide repeat; SD, sub-domain; Furin CS, furin cleavage site; FP, fusion peptide. *Total eight mutation in S1 (SD1 and 2; Furin CS) and S2 (FP, HR1) found common in all lineages.

In other structural genes, envelope protein harbors T11A in BA.2, BA.2.75, and BA.2.76 (Figure 3C). D3N was notably absent in the membrane protein of BA.2.74 and BA.2.75 (Figure 3D). The mutational signature pattern includes Q19E and A63T in M protein, T9I in E protein, and P13L, R203K, G204R, and S413R in N protein, respectively (Figure 3E). Deletion of 31–33 amino acid position in the N gene was variably present in different lineages of Omicron.

On analysis of the rest of the genome, NSP1-11 (ORF1a) was detected harboring 13 mutations comprising 12 substitutions (S135R, Q768R, T842I, S1221L, G1307L, P1640S, L3027F, T3090I, L3201F, T3255I, P3395H, and N4060S) and one deletion (Δ3575–3577). Among these mutations, important findings included the exclusive presence of S1221L and P1640S in BA.2.75 and N4060S in BA.2 and BA.2.75, while Q4060S was observed only in BA.2 and BA.2.38. In NSP12-16 (ORF1b), six mutations (P314L, G662S, T1050N, R1315C, I1566V, and T2163I) were observed in different proportions in the detected lineages. ORF3 showed two substitutions T223I and P240S in BA.2.75, while all other lineages showed only T223I. ORF6 showed D61L in all detected Omicron lineages, except BA.5.2 and BA.5.2.1, while ORF8 harbored a mutation of S84L in all lineages (Supplementary Table 1). The common signature mutation among non-structural protein of Omicron included S135R, T842I, G1307L, L3027F, T3090I, T3255I, P3395H, and Δ35753577 in ORF1a, P314L, G662S, R1315C, I1566V, and T2163I in ORF1b, T223I in ORF3a, and S84L in ORF8.

## 4. Discussion

This study has attempted to determine the prevalence of the Omicron variant and its sub-lineages circulating in Chhattisgarh. Our study identified the predominance of BA.2 and its sub-lineage (93.5%) followed by BA.5.2 and its sub-lineage BA.5.2.1 (4.6%) and B.1.1.529 (1.8%), affecting mainly the adults. Among BA.2 lineages and its sub-lineages, BA.2 (38%), BA.2.38 (32.4%), and 9.25% BA.2.75 were the predominant Omicron variant circulating in Chhattisgarh from May to July 2022. Omicron variant clade 21L (84%) was detected maximally, followed by 22B (11%) and 22D (5%). Our findings of BA.2 and BA.2.38 predominance were further supported by similar findings from the national agency named Indian SARS-CoV-2 Genomics Consortium (INSACOG) after analysis of 229,411 sequences of SARS-CoV-2 (11). An earlier study from Denmark and India has reasoned a higher fusogenicity and reproduction number (R0) of 2.445 for BA.2 predominance (12). In 2021, during the second wave of COVID-19, we reported Delta (B.1.617.2) as the chief causative agent responsible for the second wave of the COVID-19 pandemic across India along with Alpha (B.1.1.7), Kappa (B.1.617.1), and B.1 lineages in Chhattisgarh (5). In 2022, our finding of the sole presence of the Omicron variant of SARS-CoV-2 suggests that the Omicron variant has outpaced Delta and other circulatory lineages, and the predominance has been speculated to occur much earlier, probably in early 2022.

Among all the VOCs reported, Omicron’s earlier report of maximally mutating was also evident in our study, with 79 mutations detected across its genome. Of these, 46 mutations were found in the spike protein. Some of these along with a few mutations in other structural and NSP were exclusively reported in our study. Due to their omnipresence, these mutations were proposed as the Omicron-identifying signature pattern. In the spike gene of all Omicron variants, the mutational divergent hot spot region was observed predominantly in the RBD region, harboring 25 mutations, followed by 12 mutations in the NTD region (13, 14). These mutation consortia thus evidently discourage the development of any therapeutics and vaccine based on spike protein. In contrast, the poly mutational hot spot RBD and NTD regions of Omicron need to be used in diagnostic applications for the identification of individual lineages of Omicron.

Various non-synonymous mutations in spike protein have been implicated in virus infectivity, transmissibility, pathogenicity, immunological bypass, decreased neutralizing ability of monoclonal antibodies, high risk of reinfection, treatment failure, and even Omicron diagnostic detection failure (5, 15, 16). Three mutations namely I210V, F157L, and K147E located at the supersite in the NTD, along with two mutations in the RBD, Q493R and N460K, have expressively reported increased infectivity (17, 18). Earlier docking study has reported S371F, S373P, S375F, T478K, Q493R, and Q498R mutation roles in higher affinity for ACE2 receptor (4). K417N/T, G446S, S477N, E484A, F486V, and Q493R substitution rendered reduced binding of neutralizing antibody to the viral receptor to eventually help the virus in escaping innate and vaccine-induced antibody response (19). Q493R mutation was earlier reported to emerge during bamlanivimab/etesevimab cocktail treatment to confirm its role in immune evasion (20). L452R, N501Y, and D614G help the virus binding with the host ACE2 receptor resulting in increased transmission rate and infectivity (21). Q493R, Q498R, and N501Y mutations were reported to cause cross-species transmission. Notably, the D614G mutation was found to be uniformly present in all VOCs. H655Y, N679K, and P681H since adjacent to the furin cleavage site inheritance the cleavage, transmission, and developing resistance to treatment based on monoclonal antibodies (22). In contrast to the Delta variant (B.1.617.2), Omicron variant was observed with the substitutional mutations of S371F, T376A, R408S, F486V, and K417T in the RBD region and these mutations were found absent in Delta variant (22). These mutations inherit the higher transmission rate in Omicron due to higher affinity toward the ACE2 receptor (15). However, the notion “blessing in disguise” holds for humanity, as Omicron analyzed to have the absence of crucial mutation of T19R, Del 157/158 in NTD, E484K in RBD, Q15 D950N, and D1118H in S2 subunit, and modification of T19R to T19I and P681R to P981H, which all lead to reduced severity of Omicron in comparison to the Delta variant (15). Less severity of Omicron has also been evident in our study, where all the cases manifest mild upper respiratory tract illness and recover completely without requiring hospitalization.

The hot spot mutational region of RBD and NTD could plausibly explain the reason for Omicron’s high transmissibility and evading immune response (15, 23). An earlier meta-analysis has reported a 20-fold drop in neutralization antibody of convalescent sera of non-vaccinated patients with COVID-19 and a 7-fold reduction in infected vaccinated cases (15, 24). Reduced antibody titer may increase the risk of reinfection. Another study estimated the risk of reinfection with Omicron to be approximately 5.4-fold (95% CI: 4.87–6.00) higher than the Delta variant (25). The relative risks were 6.36 (95% CI: 5.23–7.74) and 5.02 (95% CI: 4.47–5.67) for unvaccinated and vaccinated cases, respectively (25). Another study reported six of the eight analyzed monoclonal antibodies were rendered ineffective against the Omicron variant (26). However, primary immunization of two doses of AstraZeneca (ChAdOx1nCoV-19) or Pfizer BioNTech (BNT162b2) vaccine provides only limited protection against the Omicron variant. Pfizer-BioNTech or Moderna (m-RNA1273) booster after completing the two-dose schedule of ChAdOx1nCoV-19 or BNT162b2 although provided substantial protection, it gradually waned over time (27). The more worrying sign in Omicron infection is a viral escape from memory T-cell response *via* CD4^+^ T-cell assisting activated naïve B cell or CD8^+^ T-cell-mediated lysis of infected SARS-CoV-2 (4).

The biological effects of the mutation in other structural and non-structural genes were also assessed. In the envelope gene, T11A was observed at the N-terminal vestibule and reported to form a type of cation channel to interfere with the binding pocket of different potential inhibitors (28). R203K and G204R in nucleocapsid protein are linked with enhanced subgenomic RNA expression and viral replication (4). Since NSP12 (RdRP) and NSP14 were reported as essential proteins required for viral replication, mutations of P314L and G662S in NSP12 and I1566V in NSP14 might confer higher replication to Omicron. This probability appeared supported by the earlier report mentioning Omicron R0 of as high as 10 and a doubling time of 2–3 days (4). The effect of other detected mutations in the envelope, membrane, nucleocapsid, and other non-structural ORF is still unknown and thus requires further research to ascertain their impact on the virus.

There could be a presumptive likelihood of BA.2.75 superseding other sub-lineages considering its adaptation of new mutations Q493R and N460K, and alteration of G339D to G339H, which all cumulatively inflicted fusogenicity to result in 44-fold higher infectivity (18). This apprehension is further supported by the earlier findings of the higher replicative ability of BA.2.75 in the Syrian hamster’s lungs in comparison with BA.2 and BA.5 (29). BA.2.75 also reported more immune evasive lineage than BA.5, especially in patients infected earlier with the Delta variant (30). It could be due to the host innate antibody response generated against L452R mutation in patients with COVID-19 infected earlier with the Delta variant was also found effective against the BA.5 variant (30). N460K, G446S, G339H, and Q493R permit BA.2.75 to escape the host effective neutralizing antibody response generated against different RBD epitopes (30). BA.2.75 sub-variant has been placed as a VOC lineage under monitoring (VOC-LUM) on 19 July 2022, together with the BA.2.12.1, BA.4, and BA.5. (2). Among them, BA.2.75 and BA.5 have already been reported from different parts of India, with BA.2.75 circulating mainly in the northern states, including Himachal Pradesh, Odisha, Haryana, Rajasthan, and Maharashtra, whereas BA.5 was reported from southern states of Tamil Nadu and Telangana (18). N460K mutation in spike, S1221L, and P1640S in ORF1a and P240S in ORF3 could be the reason for the faster transmission of BA.2.75. Thus, BA.2.75 needs to periodically monitor for any evolutionary changes for formulating countermeasures in cases of any associated outbreak.

Importantly, BA.5 lineage five omicron strains supported their likely convergent evolution. BA.5 was initially reported from the USA, European, and African countries, wherein it has replaced the other Omicron sub-lineages (31). However, at the time of reporting this study, no unprecedented upsurge of BA.5.2 and BA.5.2.1 was noticed in India. Stratifying Omicron sub-lineages divergence, the exclusive substitutions E1202Q in BA.2.43 diverge it from the other BA.2 sub-lineage. E1202Q detected in heptad repeats 2 (HR2) regions of S protein augments the viral membrane fusion with the host cell membrane (32). N460K mutations similarly confirm the divergence of BA.2.75 from BA.2.

It is worth noticing that the concept of detecting Omicron based on the absence of 69/70 deletion in the spike gene may appear to fail since 50% of BA.2, 85% of BA.2.38, and all sequences of BA.5.2 and BA.5.2.1 exhibited 69/70 amino acids to give the false negative interpretation and must be discouraged as the sole criteria of identification of Omicron.

The present study’s limitation included fewer cases and no data on vaccination and reinfection status. Nonetheless, deciphering the different Omicron lineage circulating in Chhattisgarh along with their non-synonymous mutational pattern would help in a better epidemiological understanding of the evolutionary pattern of SARS-CoV-2.

## 5. Conclusion

We conclude that different Omicron variants of clades 21L, 22B, and 22D and lineages BA.2 and BA.2.38 were predominantly circulating in Chhattisgarh with characteristic signature mutations T19I and V213G in NTD, S373P, S375F, T376A, and D405N in RBD, D614G, H655Y, N679K, and P681H at the furin cleavage site, N764K and D796K in fusion peptide, and Q954H and N969K in HR1 in spike protein. BA.2.75 appeared to emerge rapidly because of N460K mutations in spike protein, S1221L and P1640S in ORF1a, and P240S in ORF3. Thus, regular periodical genomic surveillance is needed for elucidating viral mutational insight and its effect on transmission and severity dynamics.

## Data availability statement

The data collected in this study will be provided to the only those researchers who will provide us their genuine data request endorsed by head of their institution. Such request will be presented to the institutional ethical committee of AIIMS, Raipur. On their approval, the data will be shared. The request for data should be sent to SN, negidr@aiimsraipur.edu.in.

## Ethics statement

The studies involving human participants were reviewed and approved by All India Institute of Medical Sciences Raipur (1453/IEC-AIIMSRPR/2021). The ethics committee waived the requirement of written informed consent for participation.

## Author contributions

SN, PS, KS, and AB: conceptualization. PS, KS, SN, and DS: methodology and writing—original draft preparation. KS, PS, and DS: software. SN and AB: resources. SN, PS, and KS: writing. AB and SN: supervision. All authors have read and agreed to the published version of the manuscript, and the corresponding author had final responsibility for the decision to submit for publication.
