# Supplementary material for: Mutational characterization of Omicron SARS-CoV-2 lineages circulating in Chhattisgarh, a central state of India
Source: Front Med (Lausanne). 2023 Jan 23;9:1082846. doi: 10.3389/fmed.2022.1082846 (PMC9899822; doi:10.3389/fmed.2022.1082846)
Supplement: Supplementary file 1 [file Table_1.DOCX]

**Supplementary Table 1: Non structural protein amino acid prevalence.**

| **SARS-CoV-2 genome** | **AA position** | **BA.2** | **BA.2.38** | **BA.2.43** | **BA.2.56** | **BA.2.74** | **BA.2.75** | **BA.2.76** | **BA.5.2** | **BA.5.2.1** |
| --- | --- | --- | --- | --- | --- | --- | --- | --- | --- | --- |
| **ORF1a** | **S135R** | **S135R** | **S135R** | **S135R** | **S135R** | **S135R** | **S135R** | **S135R** | **S135R** | **S135R** |
|  | **Q768R** | **Q768R** | **Q768R** |  |  |  |  |  |  |  |
|  | **T842I** | **T842I** | **T842I** | **T842I** | **T842I** | **T842I** | **T842I** | **T842I** | **T842I** | **T842I** |
|  | **S1221L** |  |  |  |  |  | **S1221L** |  |  |  |
|  | **G1307L** | **G1307L** | **G1307L** | **G1307L** | **G1307L** | **G1307L** | **G1307L** | **G1307L** | **G1307L** | **G1307L** |
|  | **P1640S** |  |  |  |  |  | **P1640S** |  |  |  |
|  | **L3027F** | **L3027F** | **L3027F** | **L3027F** | **L3027F** | **L3027F** | **L3027F** | **L3027F** | **L3027F** | **L3027F** |
|  | **T3090I** | **T3090I** | **T3090I** | **T3090I** | **T3090I** | **T3090I** | **T3090I** | **T3090I** | **T3090I** | **T3090I** |
|  | **L3201F** | **L3201F** | **L3201F** | **L3201F** | **L3201F** | **L3201F** | **L3201F** | **L3201F** |  |  |
|  | **T3255I** | **T3255I** | **T3255I** | **T3255I** | **T3255I** | **T3255I** | **T3255I** | **T3255I** | **T3255I** | **T3255I** |
|  | **P3395H** | **P3395H** | **P3395H** | **P3395H** | **P3395H** | **P3395H** | **P3395H** | **P3395H** | **P3395H** | **P3395H** |
|  | **del3575-3577** | **del3575-3577** | **del3575-3577** | **del3575-3577** | **del3575-3577** | **del3575-3577** | **del3575-3577** | **del3575-3577** | **del3575-3577** | **del3575-3577** |
|  | **N4060S** | **N4060S** |  |  |  |  | **N4060S** |  |  |  |
| **ORF1b** | **P314L** | **P314L** | **P314L** | **P314L** | **P314L** | **P314L** | **P314L** | **P314L** | **P314L** | **P314L** |
|  | **G662S** | **G662S** | **G662S** | **G662S** | **G662S** | **G662S** | **G662S** | **G662S** | **G662S** | **G662S** |
|  | **T1050N** | **T1050N** |  |  |  |  |  | **T1050N** | **T1050N** | **T1050N** |
|  | **R1315C** | **R1315C** | **R1315C** | **R1315C** | **R1315C** | **R1315C** | **R1315C** | **R1315C** | **R1315C** | **R1315C** |
|  | **I1566V** | **I1566V** | **I1566V** | **I1566V** | **I1566V** | **I1566V** | **I1566V** | **I1566V** | **I1566V** | **I1566V** |
|  | **T2163I** | **T2163I** | **T2163I** | **T2163I** | **T2163I** | **T2163I** | **T2163I** | **T2163I** | **T2163I** | **T2163I** |
| **ORF3a** | **T223I** | **T223I** | **T223I** | **T223I** | **T223I** | **T223I** | **T223I** | **T223I** | **T223I** | **T223I** |
|  | **P240S** |  |  |  |  | **P240S** |  |  |  |  |
| **ORF6** | **D61L** | **D61L** | **D61L** | **D61L** | **D61L** | **D61L** | **D61L** | **D61L** |  |  |
| **ORF8** | **S84L** | **S84L** | **S84L** | **S84L** | **S84L** | **S84L** | **S84L** | **S84L** | **S84L** | **S84L** |
